# Optical Sensing Using Dark Mode Excitation in an Asymmetric Dimer Metamaterial

**DOI:** 10.3390/s140100272

**Published:** 2013-12-24

**Authors:** Ndubuisi E. J. Omaghali, Volodymyr Tkachenko, Antonello Andreone, Giancarlo Abbate

**Affiliations:** 1 CNR-SPIN and Physics Department, University of Naples Federico II, via Cinthia, Monte S. Angelo 80126, Napoli, Italy; E-Mails: ndubuisi01@gmail.com (N.E.J.O.); abbate@na.infn.it (G.A.); 2 CNR-SPIN, via Cinthia, Monte S. Angelo 80126, Napoli, Italy; E-Mail: tkachenko@na.infn.it

**Keywords:** metamaterials, Fano resonance, dimer sensor

## Abstract

We study the presence of dark and bright modes in a planar metamaterial with a double rod unit cell introducing geometric asymmetry in rod lengths. The dark mode displays a Fano-type resonance with a sharp asymmetric profile, rendering it far more sensitive than the bright mode to slight variations of the dielectric environment. This peculiar feature may envisage the possible application of the asymmetric dimer metamaterial as an optical sensor for chemical or biological analysis, provided that the effect of material losses on the dark mode quality factor is properly taken into account.

## 1. Introduction

The flexibility in the geometry of metamaterials has enabled the tailoring of interactions between resonances in such structures, leading to exciting research possibilities such as negative index response [1], enhanced transmission [2] and electromagnetic cloaking [3]. In symmetric structures, super radiant or bright modes couple to the incident field, producing broad and lossy resonances. With the introduction of asymmetry in the metamolecule geometry, trapped or “dark” modes can be excited [4,5]. These dark mode resonances weakly couple to the free space [6,7] and therefore present high values of the quality factor Q. A Fano type resonance can result from the interference of bright and dark modes resulting in an asymmetric spectral profile [8,9]. Fano resonances caused by symmetry breaking have been reported in different structures such as double rod antenna with reduced symmetry [10–13], split rings [14,15], ring/disk systems [16], just to list a few. As far as double rod metamolecules (dimers) are concerned, there are a number of different ways to excite a quadrupolar dark plasmon mode: vertically stacking rod pairs displaced along their axis [10], introducing an additional orthogonal wire displaced from the symmetry centre [11], using oblique illumination of the symmetric dimer metasurface [12], introducing difference between rod lengths [13]. For planar metamaterials (metasurfaces) high quality resonances are challenging because of the reduced resonating volume. Nevertheless, Fano-resonant planar metallic nanostructures have the ability to strongly concentrate the electromagnetic field in small regions and increase the interaction with matter, making them promising components for the development of chemical and biological sensors [11,17,18]. In particular, infrared metamaterials can play an important rôle in biosensing and lab-on-chip applications [19,20], due to the inherent vibrational fingerprints that can be used for biomolecules identification.

For applications in the near infrared or visible wave range, the fabrication of planar metamaterials envisages the use of sophisticated electron beam lithography techniques, therefore a reduction in the complexity of their planar geometry is highly desirable, since this would decrease production time and cost dramatically. In [13] a periodical array of dimers with different rod lengths is proposed to be the most simplified structure among the abovementioned dimer based metamaterials. This metamaterial exhibits electromagnetically induced transparency, on the base of the dark and bright modes interaction. In spite of the various reports in the recent literature, only recently an analytical model—leading to a generalization of Fano formula to electromagnetic fields and lossy materials—has been proposed [21], enabling the study of these asymmetric resonances in metallic nanostructures and paving the way to their engineering. However, influence of structural parameters and intrinsic material losses on sensing properties of Fano resonance based metamaterials has not been studied properly so far.

In this work, we study numerically a planar metamaterial composed of gold nanorod dimers and report on the parametric analysis of modes excited in the IR spectrum by electromagnetic wave normally incident onto the metasurface. The dark mode, which appears due to asymmetry in the length of the gold nanorods, shows a high quality factor and a sharp dependence of its Fano resonance frequency on the environment refractive index. The dependence of the dark mode on structural parameters and its sensitivity to dielectric environment change is discussed in view of the possible application of the metamaterial under study for optical sensing, taking also into account material losses.

## 2. Metamaterial Geometry and Numerical Model

**Figure 1.**
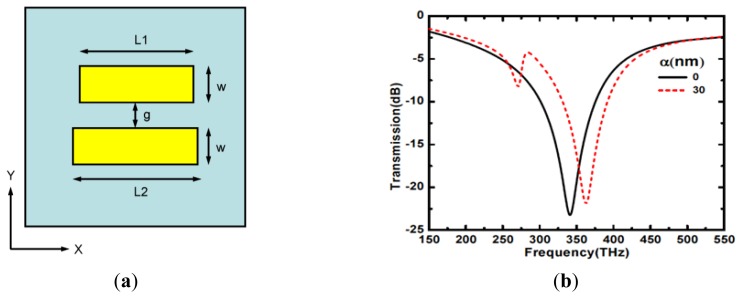
(**a**) Unit cell of the metamaterial dimer. The geometric parameters of the gold nanorods of lengths L_1_ and L_2_ are: width w = 70 nm, gap g = 50 nm. The gold thickness is 30 nm. The periodicity is 300 nm in both x and y directions. The incident wave is along the z direction. (**b**) Transmission spectra for the symmetric (solid line) and asymmetric (dashed line) dimers with α = 0 nm and α = 30 nm respectively.

The metamaterial dimer structure consists of two metallic Au nanorods on an Indium Tin Oxide (ITO)-coated glass substrate, with the ITO acting as an adhesive layer for the gold. A schematic representation of the unit cell of the dimer structure is shown in Figure 1a, with gold rods of length L_1_ and L_2_ respectively separated by a gap g = 50 nm. Both rods have equal widths w = 70 nm and thickness 30 nm. The ITO layer has a thickness of 25 nm with permittivity of 3.8. The Au permittivity at frequency *ω* is described in terms of the Drude model:

(1) $$ɛ=1-\frac{\omega_{p}^{2}}{\omega^{2}+i\omega \omega_{c}}$$

with a plasma frequency *ω_p_* = 1.37 × 10^16^ s^−1^ and a collision frequency *ω_c_* = 1.2 × 10^14^ s^−1^, to account for the scattering losses in the gold film [13,22]. We neglect the real constant offset for permittivity which appears due to the interband transitions, since it results only in a small constant frequency shift of resonances under study, without having a noticeable effect on the parameters of interest: quality factor, sensitivity to the environment, *etc*. The index of the glass substrate is taken as 1.5, the periodicity of the structure is 300 nm in both x and y directions. We employ a finite difference time domain (FDTD) solver to perform the electromagnetic simulation with wave propagation direction along the z-axis, normal to the plane containing the gold dimer and polarized along the x-axis (Figure 1a).

## 3. Results and Discussion

In our study of the resonances in the dimer structure, we define a length asymmetry α = L_2_ – L_1_, with L_2_ kept constant at 200 nm throughout. For the symmetric structure with bar lengths L_1_ = L_2_ or α = 0, we calculate the transmission spectra as shown in Figure 1b (solid line). A broad dipolar resonance occurs at about 341.2 THz and corresponds to the bright or super radiant mode which is strongly coupled to the free space. We then consider the situation in which asymmetry is introduced with unequal lengths of the bars L_1_ and L_2_ or α ≠ 0. For α = 30 nm, two resonances can be observed from the calculated spectral response, a higher frequency resonance at 362.8 THz and an associated lower frequency resonance at 270.5 THz. The higher frequency resonance of 362.8 THz is a dipole oscillation with similar characteristics like the bright mode resonance of the symmetric dimer structure (α = 0). The resonant mode at 270.5 THz, appearing because of the length asymmetry of the dimer, weakly couples to the incident field and is the so-called dark mode [23]. The interference between the bright and the dark modes results in the sharp asymmetric Fano-type profile [6,7,10,14,15,24,25] of the resonance with a characteristic dip and peak as shown in Figure 1b (dashed line).

**Figure 2.**
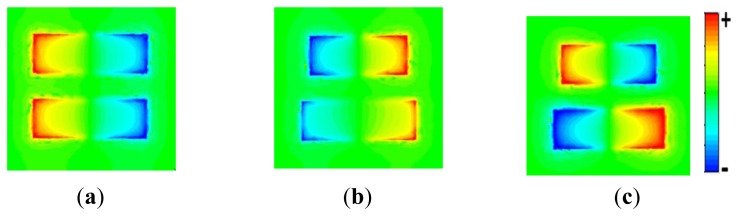
Calculated E_z_ electric field distribution for (**a**) the bright mode in the symmetric dimer at 341.2 THz, the bright (**b**) and the dark (**c**) modes in the asymmetric dimer at 362.8 and 270.5 THz respectively.

For a better insight into the nature of these resonant modes, we calculate the out-of-plane electric field (E_z_) distributions at the bright mode resonance for the symmetric dimer and at both the bright and dark mode resonances for the asymmetric dimer. E_z_ better than other field components illustrates the charge distribution inside each arm. Figure 2a shows the E_z_ distribution of the bright mode resonance at 341.2 THz in the symmetric dimer configuration depicted in Figure 1b (solid line). Here, the dimer behaves as two dipoles with parallel currents which are in-phase and symmetric. The radiation field of dipoles interferes constructively, resulting in the radiant nature of the mode. For the asymmetric dimer in Figure 1b (dashed line), the calculated field distributions are shown in Figure 2b,c for the bright and dark mode resonances, respectively. At the higher frequency resonance of 362.8 THz, a bright mode resonance similar to the dipolar mode is observed. However, at the lower frequency resonance of 270.5 THz, an antisymmetric electric field distribution is induced, as shown in Figure 2c. This corresponds to an antisymmetric current oscillation in the arms of the dimer [6,7,14,26] with very low radiation losses. The induced parallel and anti-parallel current behaviour in the asymmetric dimer of Figure 2b,c can be attributed to the bonding and anti-bonding modes in plasmon resonance hybridization [27]. The interference of this dark mode with the bright mode results in the Fano-type resonance [8,9] with an asymmetric profile, reported in Figure 1b (dashed line).

**Figure 3.**
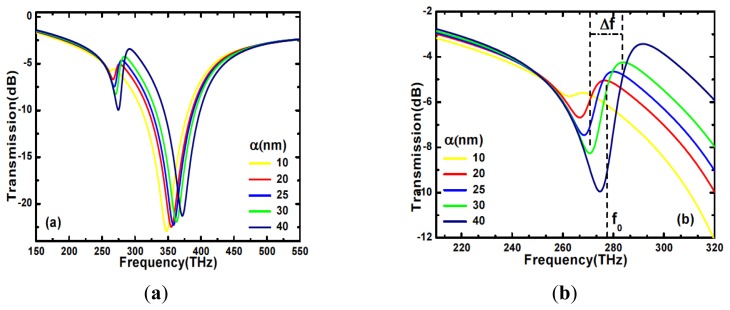
(**a**) Bright and dark mode resonances for different values of length asymmetry α. (**b**) Details of the dark mode change on asymmetry. *Δf* and *f_0_* are shown for α = 30 nm.

Having discussed the nature of these modes, we then focus our attention on the influence of asymmetry on the resonant modes. We calculate the resonance modes for different values of the length asymmetry α, from 10 to 40 nm (Figure 3a). With an increasing asymmetry, the dark mode is blue shifted and broadened as shown in Figure 3b. This can be explained since the asymmetry increases the free-space coupling and consequently the mode energy losses.

To measure the resonance broadening, we introduce for the bright mode the quality factor Q as the ratio of the dip central frequency *f_0_* and its 3-dB full width. For the Fano resonance associated with the dark mode, because of its high asymmetry we define, following [16], the quality factor as the ratio of the average frequency *f_0_* and the full width *Δf* between the peak and the antipeak (see Figure 3b).

**Figure 4.**
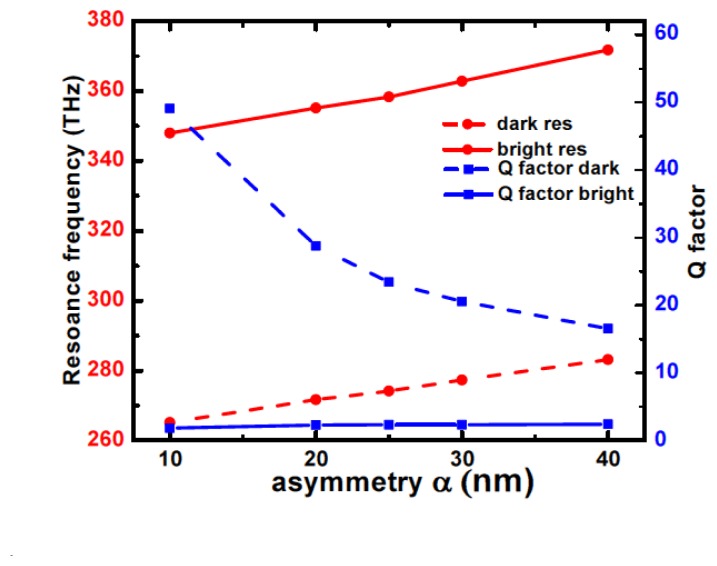
Influence of length asymmetry on resonance (red curves, left) and Q-factor (blue curves, right) for bright (solid lines) and dark (dashed lines) modes.

Therefore, for both the bright and dark mode, Q-values take into account the relative sharpness of the resonances only. For the bright mode, the resonance width broadens at a slower rate compared to the dark mode (Figure 4), as a consequence of its higher coupling to the free space, which results in an almost independent behaviour of its quality factor with length asymmetry. With increasing α, there is a corresponding blue shift of the whole spectrum due to the decrease in the total length of the two nanobars. Of course, a red shift is observed for negative values of asymmetry. Moreover, similar resonance behaviour is observed producing the same length asymmetry from either one or both ends of the nano dimer. Therefore, the asymmetry seems to be the dominant factor responsible for the excitation of the dark modes, regardless of the relative positions of the dimer arms.

## 4. Sensing with Nanodimer

**Figure 5.**
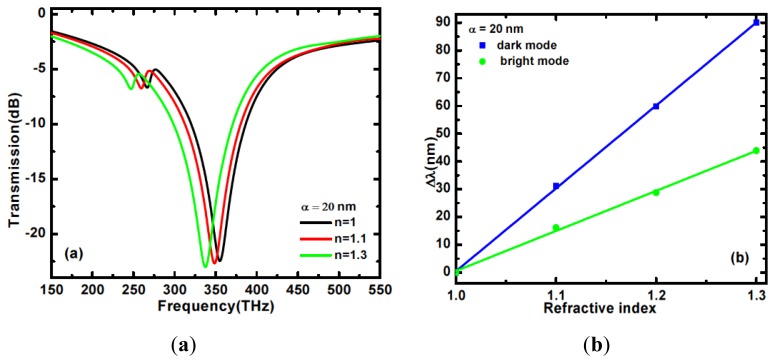
(**a**) Transmission spectra and (**b**) wavelength shift of the dark and bright resonances of the asymmetric nanodimer as a function of the surrounding dielectric for a length asymmetry α = 20 nm.

The dependence of the spectral position of resonance on the permittivity of the surrounding medium is essential for any refractive index sensing device and the sharp nature of Fano-like resonances makes them interesting for such applications. We observe that the asymmetric nano dimer is sensitive to ambient index variation and therefore opens up possibilities for applications as chemical or biological sensor. Using different *α* values of 20, 30, and 40 nm, the index of the medium surrounding the dimer is varied from n = 1.0 to n = 1.3. For all the reported values of asymmetry, a red shift of both the dark and bright resonances can be observed as the index of the surrounding medium increases. The dependence of the dark and bright mode resonances respectively on the index of the surrounding medium is shown in Figure 5a for length asymmetry of 20 nm.

**Figure 6.**
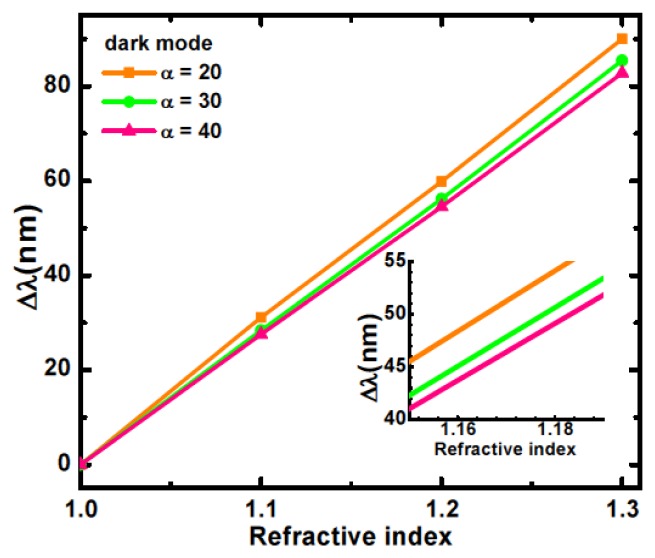
Dark mode resonance wavelength shift as a function of refractive index for length asymmetry α = 20, 30 and 40 nm respectively.

Figure 5b shows, for the same length asymmetry, the plots of the wavelength shifts in the dark and bright modes resonance of the nanostructure in a medium, varying the index of refraction. An analogous plot for the dark mode only and for different asymmetry values is presented in Figure 6. Here λ = *c/f* is taken.

The wavelength shift dependences are clearly linear and the sensitivity of the resonant wavelength to a change in the refractive index of the medium can be determined in nm/ Refractive Index Units (RIU) [28]. For the dark (bright) mode, the calculated sensitivities for length asymmetry of 20, 30, and 40 nm are 300.1 (146.3), 284.7 (147.5), and 276.1 (144.0) nm/RIU, respectively. For the Fano resonance, therefore, the sensitivity decreases as the length asymmetry increases (Figure 6). This behavior can be explained by the reduction of the electromagnetic field concentration at the metasurface with increasing length asymmetry, which is confirmed by the lowered Q value observed in Figure 4.

To compare the performance of different spectroscopic sensors, the figure of merit (FOM) appears to be a more effective parameter. FOM is defined as the sensitivity divided by the width of the resonance feature, and therefore it is inversely proportional to the smallest detectable refractive index change at the sensor output (resolution) [29]. FOM values for the dark mode at different values of length asymmetry 20, 30 and 40 nm are 7.0, 5.6, and 4.1 RIU^−1^ respectively. FOM is much lower for the bright mode, staying approximately constant at 0.4 RIU^−1^. Hence, the dark mode in the dimer appears more suitable for the development of a simple spectroscopic sensor, given the high values of both sensitivity and figure of merit. Conventional surface plasmon resonance sensors have FOM much larger, in the range of 80 [29], however at the expense of a higher complexity since they rely on the use of prisms or gratings to couple the impinging light to the surface plasmon. It is worth to emphasize that provided FOM values in the asymmetric dimer configuration are larger than those for an array of spherical gold nanoparticles with comparable dimensions (particle diameter and lattice period) [30].

**Figure 7.**
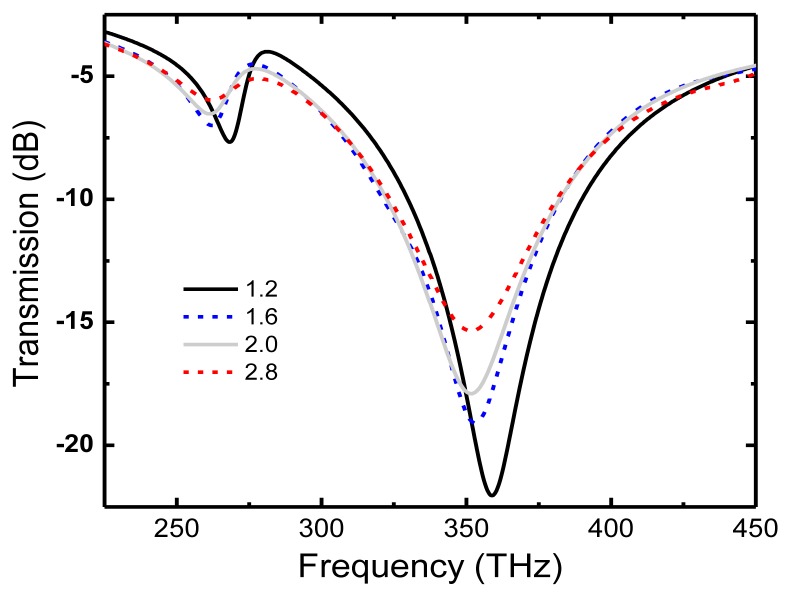
Dark and bright mode resonances for collision frequency varying from 1.2 to 2.8 × 10^14^ s^−1^ and α = 30 nm.

The value for the collision frequency we used till now is what is usually found in literature for gold [13–18,22]. However, realistic values for evaporated thin films can be several times as much, because of the granular structure of the film itself [31], resulting in an increase of the intrinsic material losses. Therefore, we show in Figure 7 the dark and bright mode resonances behavior for values of the Au collision frequency varying from 1.2 to 2.8 × 10^14^ s^−1^.

**Figure 8.**
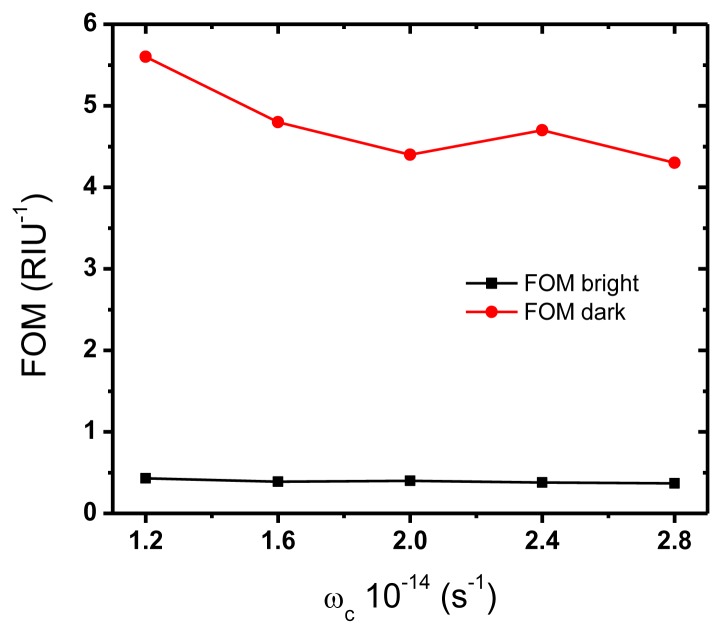
FOM values varying the collision frequency from 1.2 to 2.8 × 10^14^ s^−1^ for the dark (red circles) and bright (black squares) modes respectively (α = 30 nm). Continuous lines are guide-to-the-eye only.

As expected, both resonances broaden and decrease the contrast with an increase in the collision frequency value. However, the dark mode is much more affected by material losses than the bright one because of its inherent non-radiative nature. The way FOM values change with varying collision frequency is presented in Figure 8 for both modes and for length asymmetry α = 30 nm.

The simple geometry of the present metamolecule allows us to easily identify a small number of structural parameters that are particularly vulnerable to fabrication imperfections and to investigate their impact on the spectral response of the dimer structure. This is rather more complicated in case of other asymmetric structures like split ring and ring/disc based systems. Uncertainty of structural parameters such as arm length and width, lateral distance and relative longitudinal shift between the bars is simply proportional to the lateral accuracy of electron beam lithography. We assume this uncertainty be ±5 nm. In a real world, these parameters may randomly change for different metamolecules in the overall structure. However, since e.m. simulation over an array of many dimers is computationally very demanding, we only studied the optical response for the single unit cell changing one-by-one the single structural parameters in the range (−5, +5) nanometers.

Results of these simple simulations show that the most critical parameter affecting the response of the dark mode is the arm length. Actually ±5 nm uncertainty causes about 9 THz spread of the resonance frequency. Inhomogeneous broadening of the resonance curve, caused by random change of bar lengths, must be therefore less than 9 THz, corresponding to 30% decrease in FOM without affecting sensitivity. The other structural parameters are less critical: width and gap variations only slightly change the resonance frequency (3 THz at most), whereas shifting longitudinally the arms produces less than 1 THz change. The prominent rôle played by the arm length, in comparison to the other parameters, clearly originates from the fact that longitudinal oscillations of charges are responsible for the observed plasmon resonances in the dimer.

## 5. Conclusions

The excitation of dark modes due to the effect of symmetry breaking in the plasmonic dimer nanostructures has been studied. These trapped modes display Fano-type resonance, because of their interference with the bright modes. The sensitivity of the dark resonances to the external dielectric environment in a simple planar nanodimer metamaterial envisages the possible application of this structure as a chemical or biological sensor. The introduction of asymmetry in the length of the nanorod dimer is the dominant factor in the excitation of the dark modes and is independent of the way it is generated, rendering this kind of sensor very robust to fabrication defects or other structural imperfections. Nevertheless, because of the non-radiative nature of the dark mode, intrinsic material losses have to be taken into account for the design of Fano-resonance based optical sensors.
